# A drug-induced hypotensive challenge to verify catheter-based radiofrequency renal denervation in an obese hypertensive swine model

**DOI:** 10.1007/s00392-020-01764-0

**Published:** 2020-11-02

**Authors:** Lucas Lauder, L. Boyce Moon, Catherine A. Pipenhagen, Sebastian Ewen, Jeffrey M. Fish, Renu Virmani, James A. Jensen, Michael Böhm, Felix Mahfoud

**Affiliations:** 1 Klinik für Innere Medizin III, Kardiologie, Angiologie und Internistische Intensivmedizin, Universitätsklinikum des Saarlandes, Saarland University, Kirrberger Str. 100, Geb. 41.1 (IMED), 66421 Homburg, Saar Germany; 2 Abbott, Inc, Plymouth, MN USA; 3 CVPath Institute, Gaithersburg, MD USA; 4 Institute for Medical Engineering and Science, MIT, Cambridge, MA USA

**Keywords:** Hypotensive challenge, Arterial hypertension, Renal denervation, Renal arterial microanatomy

## Abstract

**Objective:**

Sham-controlled trials provided proof-of-principle for the blood pressure-lowering effect of catheter-based renal denervation (RDN). However, indicators for the immediate assessment of treatment success are lacking. This study sought to investigate the impact of RDN on renal renin arteriovenous difference (renal renin AV-Δ) following a hypotensive challenge (HC).

**Methods:**

Twelve hypertensive Ossabaw swine underwent either combined surgical and chemical (*n* = 3) or catheter-based RDN (*n* = 9). A telemetry monitor was implanted to acquire hemodynamic data continuously. Before and after RDN, a sodium nitroprusside-induced HC was performed. Renal renin AV-Δ was calculated as the difference of plasma renin concentrations drawn from the renal artery and vein.

**Results:**

In total, complete renal renin AV data were obtained in eight animals at baseline and six animals at baseline and 3 months of follow-up. Baseline renal renin AV-Δ correlated inversely with change in 24-h minimum systolic (− 0.764, p = 0.02), diastolic (*r* = − 0.679, *p* = 0.04), and mean (*r* = − 0.663, *p* = 0.05) blood pressure. In the animals with complete renin secretion data at baseline and follow-up, the HC increased renal renin AV-Δ at baseline, while this effect was attenuated following RDN (0.55 ± 0.34 pg/ml versus − 0.10 ± 0.16 pg/ml, *p* = 0.003). Renin urinary excretion remained unchanged throughout the study (baseline 0.286 ± 0.187 pg/ml versus termination 0.305 ± 0.072 pg/ml, *p* = 0.789).

**Conclusion:**

Renin secretion induced by HC was attenuated following RDN and may serve as an indicator for patient selection and guide successful RDN procedures.

**Electronic supplementary material:**

The online version of this article (10.1007/s00392-020-01764-0) contains supplementary material, which is available to authorized users.

## Introduction

Catheter-based renal denervation (RDN) has been introduced to reduce blood pressure (BP) and sympathetic activity by interrupting afferent and efferent sympathetic nerve signaling [1]. The results of the most recent randomized, sham-controlled trials provided biological proof-of-principle for the BP-lowering efficacy and safety of ultrasound- and radiofrequency (RF)-based RDN in hypertensive patients with and without concomitant antihypertensive medication [2–4].

Since the BP response following RDN is subject to considerable interindividual variation, the procedure remains a black box without any direct read-out of successful renal nerve ablation [5]. Various determinants for future BP response have been explored previously with at the best equivocal success [1, 6]. The renal sympathetic nervous system’s activation mediates vasoconstriction, sodium retention, and renin secretion [7]. In preclinical studies, controlled hypotension with sodium nitroprusside resulted in elevations of renin secretion [8]. Therefore, it is plausible that medically induced hypotension might increase renal sympathetic nerve activity and increase renal renin arteriovenous difference (renal renin AV-Δ) [7]. Conversely, the interruption of renal sympathetic nerve signaling might attenuate renin secretion following a sodium nitroprusside-induced hypotensive challenge (HC). In this study, we aimed at investigating the predictive value of a sodium nitroprusside-induced HC before and after RDN on renal renin AV-Δ, kidney norepinephrine, and future BP change in a hypertensive swine model.

## Methods and materials

**Fig. 1:**
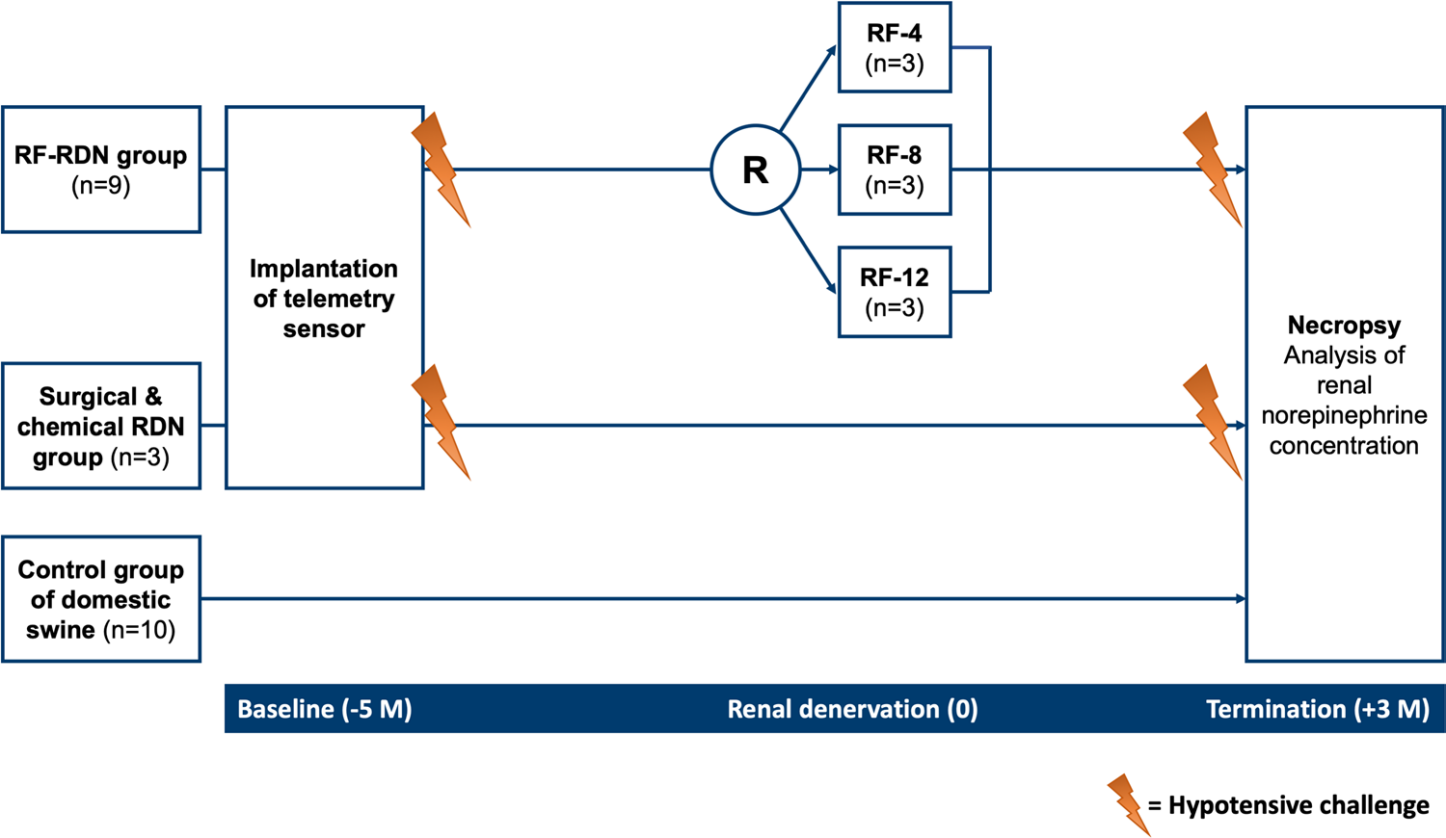
Study design. A total of 12 Ossabaw swine underwent renal denervation. Nine animals underwent minimally invasive catheter-based renal denervation (RDN) using a multielectrode radiofrequency (RF) catheter (EnligHTN Renal Artery ablation Catheter, Abbott, MN, USA) with four (RF-4), eight (RF-8) or 12 (RF-12) ablation lesions. Three Ossabaw swine had a combined surgical and chemical RDN. Five months (– 5 M) before RDN, a telemetry device was implanted. The hypotensive challenge was performed 5 months before (− 5 M) and 3 months after (+ 3 M) RDN. 10 domestic swine served as controls for comparisons of renal tissue norepinephrine concentrations between treated and untreated animals. *M* months, *RDN* renal denervation, *RF* radiofrequency

The study methodology and the primary BP outcomes have been published elsewhere [9]. In this predefined sub-study, a total of nine Ossabaw swine were randomly allocated to catheter-based RF RDN receiving either bilaterally four (RF-4), eight (RF-8), or twelve (RF-12) RF-ablation lesions (Fig. 1). The second group of three Ossabaw swine underwent combined surgical and chemical RDN. At baseline (5 months before RDN), a telemetry device was surgically implanted during anesthesia in all 12 Ossabaw swine to monitor and acquire hemodynamic data continuously throughout the study period. Further, at baseline and before termination (3 months after RDN), the treatment group animals underwent an HC with blood sampling immediately before (pre-HC) and after HC (post-HC). Urine samples were drawn after the HC. At termination, all swine were sacrificed to measure renal norepinephrine concentrations and for histopathological examinations. Ten domestic swine served as controls for comparison of kidney norepinephrine (NEPI) concentrations of treated and untreated animals. The study was conducted in accordance with the Guide for the Care and Use of Laboratory Animals under approved institutional animal care and use committee-approved protocol.

### Hypertensive model

**Fig. 2:**
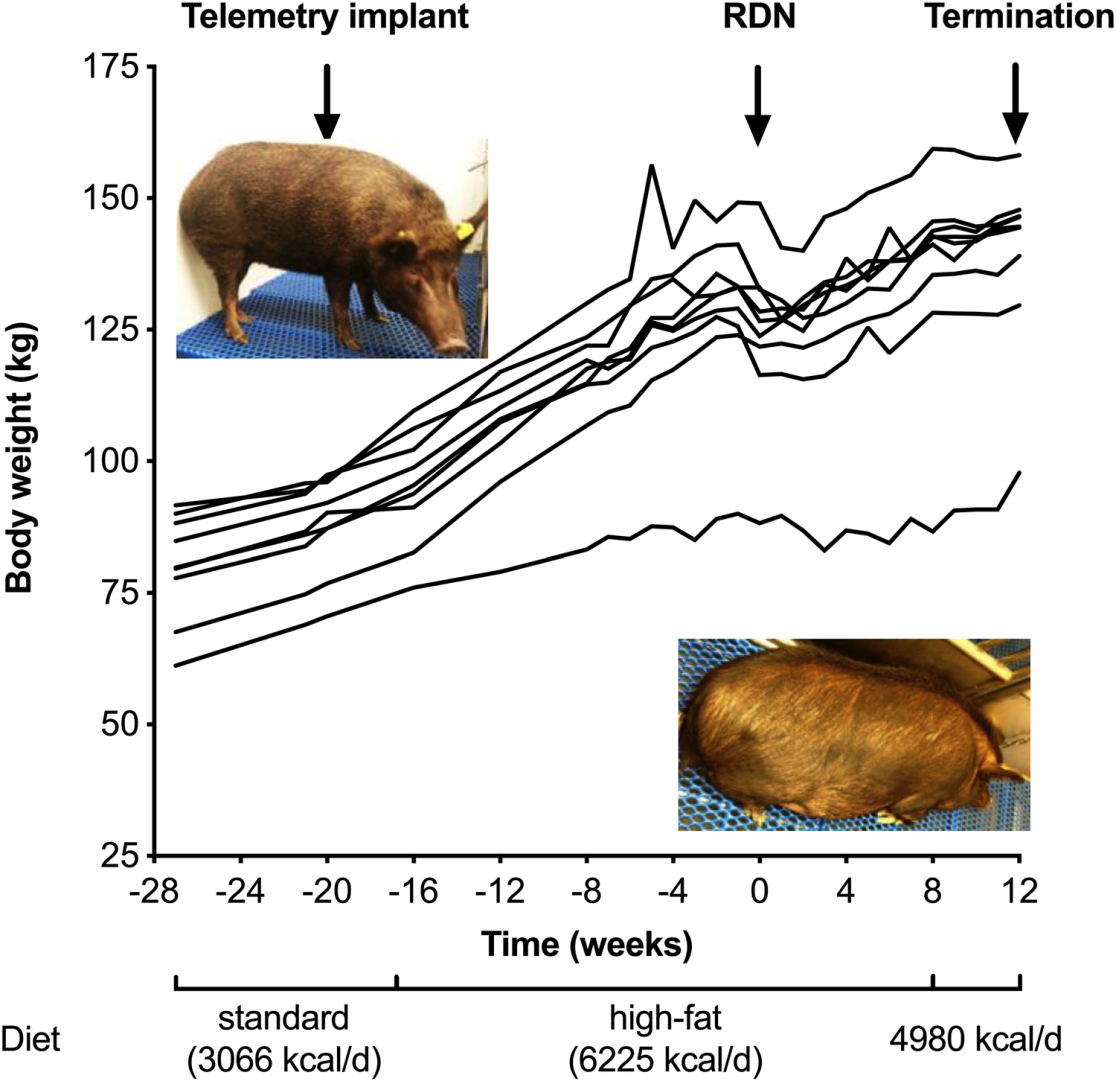
Hypertensive model development. The figure shows the change in bodyweight for all animals with complete follow-up. The hypertensive model development has been described previously in detail [9]. At the start of the study, all swine were on a standard diet. After the telemetry implant procedure and 21-day recovery period, a high-fat diet was initiated, causing a significant increase in bodyweight, cholesterol, and blood glucose levels. At the time of renal denervation (RDN) therapy, all animals had developed arterial hypertension. *RDN* renal denervation

All animals fed the same high-calorie diet causing an increase in bodyweight (+ 51.8 ± 13.3 kg, *p* < 0.001) (Fig. 2), cholesterol (+ 2.547 ± 1.692 mmol/l, *p* = 0.002), and blood glucose levels (+ 1.248 ± 1.448 mmol/l, *p* = 0.032) throughout the study [9]. Further, hypertension was developed in all pigs (systolic/diastolic BP at baseline 142.8 ± 5.2/107.6 ± 5.2 versus 169.5 ± 6.9/128.3 ± 5.7 mmHg at RDN, *p* < 0.001). Renal function remained unchanged during the study (creatinine at baseline 0.027 ± 0.01 mmol/l versus 0.026 ± 0.011 mmol/l at termination, *p* = 0.5).

### Telemetric hemodynamic monitoring

In all animals, implantable telemetry devices (PhysioTel Digital model L11; Data Sciences Int.[DSI], St Paul, MN, USA) were used to monitor and collect hemodynamic parameters. The transmitters were implanted in an intermuscular pocket in the neck area using standard surgical techniques and covered with surgical mesh to facilitate tissue healing. The systemic BP catheter was tunneled and inserted into the carotid artery close to the carotid bifurcation. The negative ECG biopotential lead was introduced into the jugular vein and advanced caudally to place the distal tip at the junction of the superior vena cava and right atrium. The distal end of the positive ECG lead was tunneled subcutaneously and secured at the left lateral costal arch level to acquire an ECG in lead II configuration. For the analyses of hemodynamic data, we calculated the 24-h, daytime (0600–1800), and nighttime (1800–0600) average BP and heart rate values. Furthermore, the minimum and the maximum values during the 24-h recording period were documented.

### Hypotensive challenge

During anesthesia, an HC was induced by a 5-min intravenous infusion of sodium nitroprusside (2 μg/kg of bodyweight per min, diluted in glucose 5%), which reduces pre- and afterload as it releases nitric oxide, causing arterial and venous vasodilatation. The HC was performed 5 months before RDN during telemetry implantation and 3 months after RDN prior to termination.

### Renal denervation

Nine animals underwent minimally invasive catheter-based RDN using a multielectrode RF catheter (EnligHTN Renal Artery ablation Catheter, Abbott, MN, USA), whereas three animals were assigned to combined surgical and chemical RDN. For catheter-based RDN, an 8 Fr sheath was placed in the femoral artery under sterile conditions. Selective angiography and quantitative vascular analysis were performed on both sides to assess anatomical eligibility for RDN. Appropriate renal artery anatomy included main renal arteries between 4 and 8 mm in diameter and with sufficient length in the absence of accessory renal arteries. The RDN catheter was introduced into the main renal artery via a guide catheter and was connected with the dedicated generator (model 100,078,171). The animals bilaterally received four (*n* = 3), eight (*n* = 3), or twelve (*n* = 3) ablations. Before and 5 min post-treatment, nitroglycerin was injected into the renal artery. A post-treatment angiography was performed before removing the sheath. Three animals underwent combined surgical and chemical RDN requiring median laparotomy. First, all visible nerves surrounding the renal arteries were disrupted. Then, the top layers of the renal artery adventitia (2–4 mm) were stripped, and finally, the surface was covered with a phenol/ethanol solution (10–20%) for 10–15 min causing neurolysis.

### Blood and urine analysis

Blood samples from the renal artery and vein were drawn immediately before and after the 5-minute intravenous infusion of sodium nitroprusside. Urinary catheterization was used to collect urine samples after the HC. Renin concentrations were measured using a porcine renin ELISA kit (NeoBiolab, Cambridge, MA, USA). The estimated renal renin AV-Δ was calculated by subtracting the arterial from venous renin concentration.

### Necropsy

At the end of the 8-month study period, a necropsy was performed in every animal by a Diplomat, American College of Veterinary Pathologists, as described previously in detail [9].

### Kidney tissue norepinephrine concentration

The left and right kidney were removed, and after examination, the renal tissue was immediately placed into liquid nitrogen at − 20 °C and homogenized. A sample of the homogenate was thawed and extracted with ammonium acetate buffer. The extracts were purified by a multisolvent solid-phase extraction (SPE) purification process. For analysis, the eluate from the SPE cartridges was injected into high-performance liquid chromatography (HLPC) instrument with electrochemical detection (ECD) system.

### Histopathological analysis

Five renal arteries were selected for the evaluation of nerve distribution. The renal arteries were cut into equal transverse segments (3–4 mm) before being fixed and embedded in paraffin. All sections were processed using alcohols and xylenes, cut on a rotary microtome at 5 µm, and then stained with hematoxylin and eosin, modified Movat Pentachrome, and neurofilament protein (NFP). All slides were imaged with the Axio Scan.Z1 slide scanner equipped with Zeiss Efficient Navigation (ZEN) 2012 software (Zeiss, Jena, Germany). Measurements were performed with HALO software (Indica Labs, Corrales, MN, USA).

### Statistical analyses

Data are presented as mean ± standard deviation (SD) and numbers (%) unless otherwise specified. A two-tailed *p* value < 0.05 was considered to be statistically significant. The Mann–Whitney *U*, Wilcoxon, and Kruskal–Wallis test by ranks were used for non-normal data. Pearson’s correlation coefficient was calculated for baseline renal renin AV-Δ and change in systolic, mean, and diastolic BP. STATA, version 16.1 (StataCorp LLC, College Station, TX, USA) and Minitab, Version 17.0.1 (Minitab Inc., State College PA, USA) were used for statistical analysis. Graphs were created using GraphPad Prism, version 8.0.1 (GraphPad Software, San Diego, CA, USA).

## Results

### Hemodynamic analysis

**Fig. 3:**
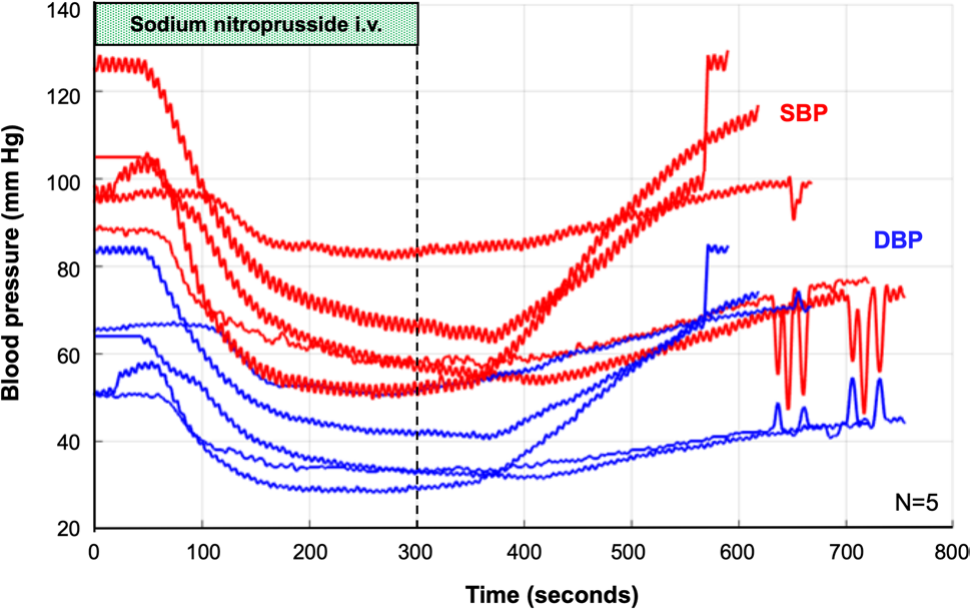
Example of blood pressure recordings during a hypotensive challenge. The figure shows an example of blood pressure recordings during a hypotensive challenge of five representative animals at baseline (prior to RDN). The hypotensive challenge was induced by a 5-minute intravenous infusion of 2 μg sodium nitroprusside per kg of bodyweight per minute. The red and blue lines depict the systolic and diastolic blood pressure values, respectively. *DBP* diastolic blood pressure, *SBP* systolic blood pressure

The hemodynamic changes of the RF-based RDN group have been described previously in detail [9]. In brief, BP significantly dropped 45 days post-RDN. At termination, the 24-h BP returned nearly to baseline values. Given the strong association between BP and bodyweight (*R*^2^ = 0.87, *p* < 0.001) and the fact that the animals continued to gain weight following RDN (+ 11.4 ± 6.2 kg, *p* < 0.001), group mean systolic and diastolic BP were normalized by weight. The most pronounced systolic (− 12.4 mmHg) and diastolic (− 11.2 mmHg) BP reduction were observed during the most-active-hour period. The hemodynamic data, including those who underwent combined surgical and chemical denervation, are summarized in Table 1. Weight-normalized BP decreased through 45 days and 3 months post-RDN. The drug-induced HC acutely lowered systolic and diastolic BP by 44.9 ± 19.8/28.3 ± 11.2 mmHg on average. Figure 3 shows an example of BP recordings during an HC in five animals at baseline.

**Table 1:** 24-h blood pressure recordings at pre-renal denervation baseline, 45 days, and 90 days post-renal denervation in the nine animals which completed 90 days of follow-up

Mean 24-h blood pressure	Pre-RDN	45 days post-RDN		90 days post-RDN	
		Value	*p* value^a^	Value	*p* value^a^
SBP, mmHg	167.6 ± 14.1	160.4 ± 18.3	0.031	170.5 ± 18.67	0.398
SBP/weight^b^, mmHg/kg	1.34 ± 0.18	1.24 ± 0.17	0.008	1.24 ± 0.05	0.003
MAP, mmHg	147.0 ± 12.7	140.2 ± 15.6	0.011	148.4 ± 16.2	0.598
MAP/weight^b^, mmHg/kg	1.17 ± 0.16	1.09 ± 0.15	0.002	1.08 ± 0.13	0.001
DBP, mmHg	127.6 ± 11.8	121.1 ± 14.2	0.01	127.9 ± 14.9	0.872
DBP/weight^b^, mmHg/kg	1.0 ± 0.13	0.94 ± 0.13	0.001	0.93 ± 0.11	< 0.001

Values are mean ± standard deviation (SD) and interquartile ranges (IQR)

*DBP* diastolic blood pressure, *RDN* renal denervation, *SBP* systolic blood pressure

^a^*p* values for comparison of mean 24-h blood pressure at 45 days and 90 days post-RDN versus pre-RDN were calculated utilizing a paired *t* test

^b^Bodyweight-normalized blood pressure (mmHg/kg)

### Blood and urinary renin levels

**Fig. 4:**
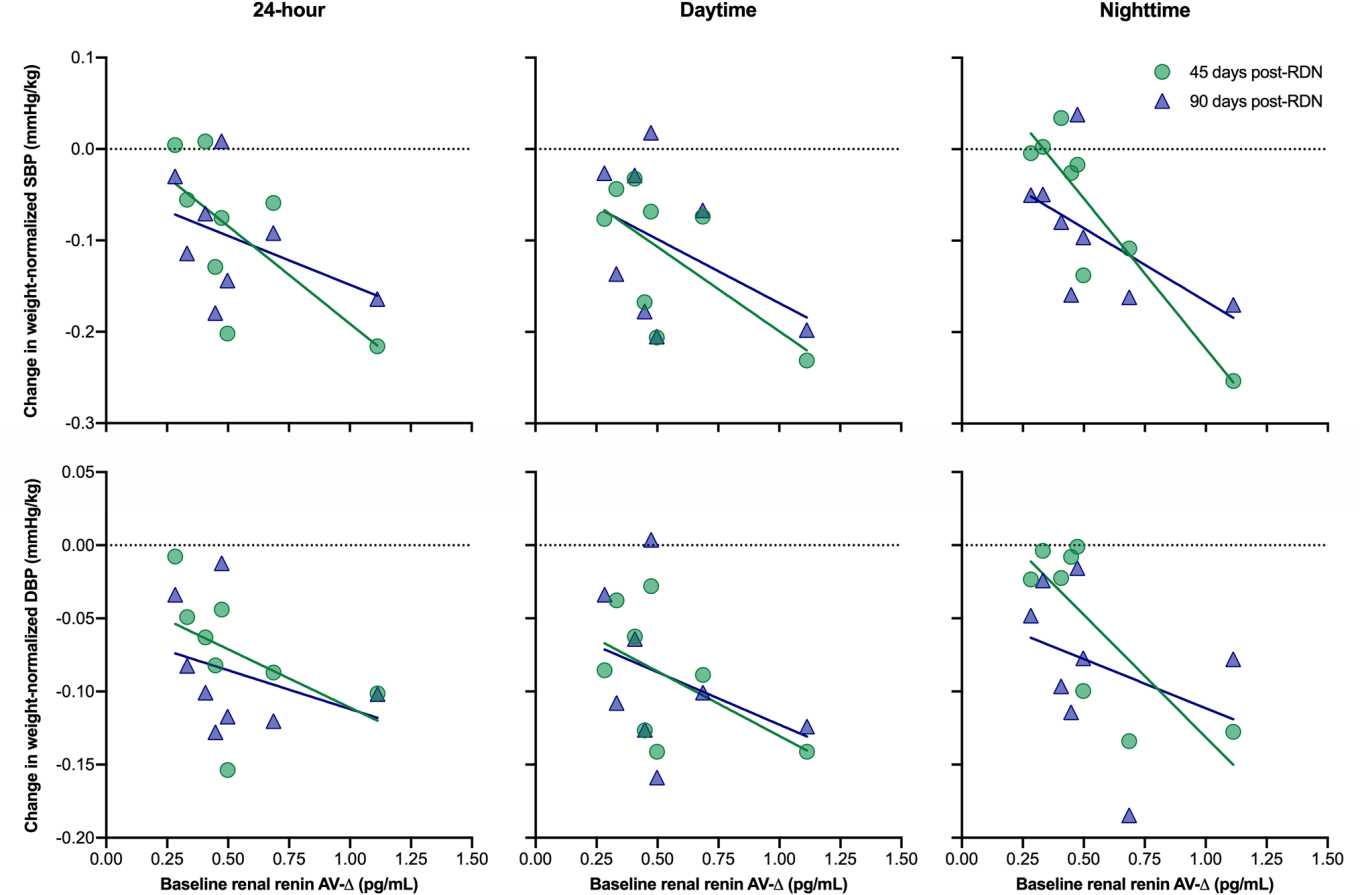
Association of baseline renal renin AV-Δ and weight-normalized blood pressure at 45 days and 90 days post-RDN

**Fig. 5:**
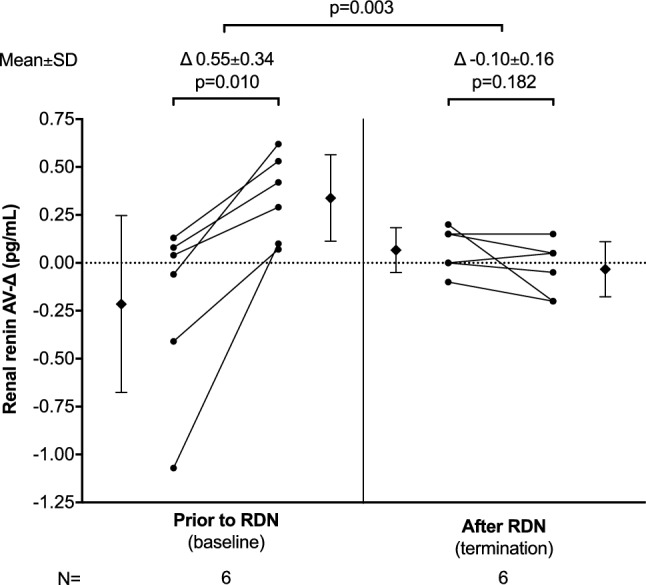
Renal renin arteriovenous difference following a hypotensive challenge before and after RDN. At baseline (prior to RDN) and before termination (after RDN), the treatment group animals underwent a hypotensive challenge. Blood samples from the renal artery and vein were drawn before and after the hypotensive challenge. Renin concentrations were measured using a porcine renin ELISA kit (NeoBiolab, Cambridge, MA, USA) and renal renin arteriovenous difference (AV-Δ) was calculated by subtracting the arterial from venous renin concentration. Complete renin secretion data was available in 6 animals at baseline and termination

The individual renin secretion data are provided in the supplemental table S1. Table 2 shows the correlation between baseline renal renin AV-Δ and the change in BP at 45 days and 90 days post-RDN in all eight animals with complete renin data at baseline that completed follow-up. While there was no significant correlation between baseline renal renin AV-Δ and mean 24-h or daytime BP, there was a strong correlation between baseline renal renin AV-Δ and change in weight-normalized nighttime systolic (*r* = − 0.846, *p* = 0.008) and diastolic BP (*r* = − 0.769, *p* = 0.026) after 45 days (Fig. 4). As BP was highly susceptible to external influences (e.g., illumination, feeding), we documented and analyzed the minimum BP values during the 24-h BP recording period. Baseline renal renin AV-Δ correlated significantly with the change in the minimum values of systolic (− 0.764, *p* = 0.02), mean arterial (*r* = − 0.663, *p* = 0.05), and diastolic (*r* = − 0.679, *p* = 0.04) BP at 90 days. For six animals, complete renin secretion data were available at baseline and termination. In these, the HC caused a significant increase in renal renin AV-Δ at baseline, whereas the renin secretion was attenuated post-RDN (0.55 ± 0.34 pg/ml versus − 0.10 ± 0.16 pg/ml, *p* = 0.003) (Fig. 5). Urinary renin secretion remained unchanged throughout the study period (0.26 ± 0.16 pg/ml at baseline versus 0.31 ± 0.07 pg/ml at termination, *p* = 0.47).

**Table 2:** Correlation between baseline renal renin AV-Δ and change in BP at 45 days and 90 days post-RDN

Weight-normalized BP^a^	*N*	Baseline renal renin AV-Δ			
		45 days post-RDN		90 days post-RDN	
		*r*	*p* value	*r*	*p* value
24-h SBP/weight, mmHg/kg	8	– 0.664	0.073	– 0.431	0.286
24-h DBP/weight, mmHg/kg	8	– 0.483	0.226	– 0.332	0.422
Daytime SBP/weight, mmHg/kg	8	– 0.632	0.093	– 0.424	0.296
Daytime DBP/weight, mmHg/kg	8	– 0.524	0.183	– 0.353	0.391
Nighttime SBP/weight, mmHg/kg	8	– 0.846	0.008	– 0.589	0.124
Nighttime DBP/weight, mmHg/kg	8	– 0.769	0.026	– 0.327	0.429

^a^Bodyweight-normalized blood pressure (mmHg/kg)

### Renal tissue norepinephrine concentrations

**Fig. 6:**
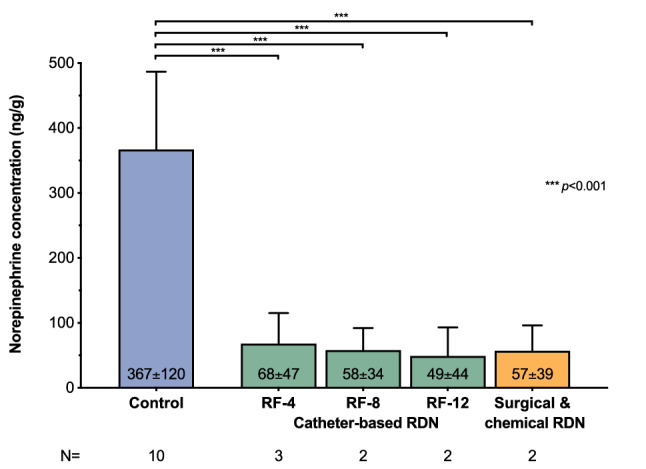
Renal tissue norepinephrine concentrations at 90 days post-RDN. At the end of the 8-month study period, a necropsy was performed in all animals that completed 90 days of post-RDN follow-up (*n* = 9) and ten domestic swine which served as controls for comparisons of treated and untreated animals. Renal tissue norepinephrine concentrations were assessed for both the left and right kidney in every animal using high-performance liquid chromatography (HPLC). *RF* radiofrequency

RDN resulted in a significant reduction of kidney NEPI concentrations compared with the control group, irrespective of the method or number of bilateral RF lesions (for every treatment subgroup versus control group *p* < 0.001, Fig. 6). Although NEPI concentrations following RDN were numerically lower in groups with more bilateral ablations, the in-between-group difference did not meet statistical significance (*p* = 0.91).

### Histopathology

A total of 1263 nerves from 47 sections (7 proximal, 5 middle, 15 distal, and 20 post-bifurcation sections) were identified (supplemental table S2). 397 nerves were considered large as 298 and 99 had a diameter ≥ 100 µm and ≥ 200 µm, respectively. The number of nerves was highest in the superior and lowest in the posterior region (supplemental table S2). Mean minimal diameter of nerves was similar among proximal, middle, distal and post-bifurcation segments (*p* = 0.15) (supplemental table S3) but was smaller in nerves located close to the renal artery lumen compared to nerves located further away in proximal sections (*p* = 0.002). In the proximal segment, the number of large nerves (diameter ≥ 100 µm) was highest in regions with > 6 mm distance from the arterial lumen, whereas, in the more distal segments, the number of large renal nerves is the greatest close to the arterial lumen. No nerve with a diameter ≥ 200 µm was identified within a 2-mm distance from the arterial lumen in the proximal segment.

### Early deaths

Three animals died from sudden cardiac death within 3 days after RDN. These early deaths occurred irrespective of the method used in three different treatment subgroups (combined surgical and chemical, RF-8 and RF-12). The affected animals were very unhealthy, had severe hypertension, and metabolic derangements at the time of treatment.

## Discussion

The present study provides important information and enhances our understanding of catheter-based renal sympathetic denervation. In this cohort of hypertensive Ossabaw swine, drug-induced HC significantly increased renin secretion, which was attenuated after RDN. Notably, a higher renal renin AV-Δ at baseline was associated with a more pronounced reduction in weight-normalized 24-h mean arterial and diastolic BP at follow-up.

An overactive sympathetic system plays a pivotal role in the pathophysiology of hypertension and associated comorbidities [10]. Efferent sympathetic renal nerves can increase renin secretion, stimulate sodium reabsorption, and reduce renal blood flow while afferent sympathetic nerves influence central sympathetic nervous system activity [7, 11]. RDN was introduced to modulate renal sympathetic nerve signaling. Recent sham-controlled trials have provided biological proof-of-principle for the BP-lowering efficacy of catheter-based RDN. Although revised treatment approaches and different treatment modalities (i.e., RF and ultrasound) have been used in these studies, BP response following RDN underlies a considerable interindividual variability for various reasons, with up to 30% of patients showing limited BP-lowering effects [3, 4, 12]. In some patients, inadequate BP response might be caused by insufficient or incomplete targeting of sympathetic nerve fibers.

One of the major unresolved issues is how treatment success can be monitored intraprocedurally. Up until today, no feedback is provided to the interventionist during the procedure and performing too few ablations per artery or targeting the wrong arterial segments could result in high variability of BP reduction or even in a complete adverse outcome. Several biomarkers have been investigated but did not provide relevant prognostic information for future BP response [13–15]. Renal nerve stimulation, which acutely raises BP, has yet not been investigated in larger, randomized, controlled trials [16].

Herein, we investigated the effect of catheter-based RDN on renal renin AV-Δ induced by a standardized HC. Renin is an aspartyl protease secreted by renal juxtaglomerular cells and is the rate-determining step in the activation of the renin-angiotensin system, which is responsible for maintaining adequate BP and fluid balance [17]. The primary stimuli for renin synthesis and secretion are increased renal sympathetic stimulation, low renal perfusion pressure, and decreased filtered sodium in the kidney’s distal tubule [17, 18]. Circulating renin and angiotensin-converting enzyme cleave amino acids from angiotensinogen in a two-step reaction to form angiotensin II [17, 18]. The HC induced by injection of nitroprusside significantly lowered BP (Fig. 3), which might have caused renal sympathetic nerve activation and thereby increased renal renin AV-Δ before RDN. Following the RDN procedure, however, this increase in renal renin AV-Δ was attenuated, indicating a successful interruption of renal sympathetic nerve traffic, which has also been confirmed by a 90% reduction of NEPI kidney concentration post-mortem. Of note, there was no difference in kidney tissue NEPI reduction between surgical/chemical and catheter-based RDN. Attenuated renal renin AV-Δ might represent a novel indicator of sufficient renal sympathetic nerve disruption and help guide RDN procedures. Importantly, the renin secretion pattern during HC predicted future BP change in this hypertensive swine model. This pattern is interesting because a common feature of all studies in RDN is the variability of the treatment effects. Therefore, the identification of patients with a high likelihood of response to RDN remains a critical unmet need. Once our findings have been confirmed in humans, the profile of renin release triggered by standardized HC before RDN may help to identify patients with renal sympathetic activity involved in the BP regulation, and thereby potential candidates for RDN treatments.

The histopathological analysis of the renal arteries showed a conical distribution of large renal nerves (diameter ≥ 100 µm). While large nerves were typically located close to the arterial lumen in distal renal artery segments, they were further away from the lumen in proximal segments. These findings are consistent with the results of animal and human autopsy studies [19, 20]. In a porcine model, combined main renal artery and branch ablation resulted in significantly greater reductions in renal norepinephrine levels and axon density than treatment of the main renal artery only. While two clinical studies in humans confirmed that BP-lowering was more pronounced following combined main renal artery and branch ablation compared with main renal artery ablation only [21, 22], there was no difference in BP reduction between patients with ablations of the main renal artery only and those with additional side branch ablation in the RADIOSOUND-HTN trial [23]. As the penetration depth of RDN catheter systems remains device-specific, and renal nerves are located closer to the lumen with increasing distance from the ostium, the treatment of distal renal artery segments may improve the completeness of renal nerve disruption, at least with commercially available RF systems. Treating patients, according to renal innervation patterns, may increase the likelihood of treatment success following RDN. However, given the vast diversity of renal nerve distribution and microanatomy differences at the treatment site, an individualized, guided approach would be desirable [24]. Renal renin AV-Δ induced by HC may represent an interesting new tool in this context, which should be investigated in future clinical studies.

### Limitations

As with any other animal model the results might not be exactly translated to a human population although the Ossabaw pig renovascular anatomy is very similar to that of humans and is considered the preferred animal model for RDN [25, 26]. All animals were very unhealthy at the time of treatment and three animals died within three days after RDN from sudden cardiac death. These deaths occurred irrespective of the method used and are a known phenomenon in the obese Ossabaw swine model. This study aimed to investigate the predictive value of the renal renin AV-Δ following a sodium nitroprusside-induced HC and future BP change. Although BP significantly dropped 45 days post-RDN, the 24-h BP returned nearly to baseline values at termination. Given the strong association between BP and bodyweight, the increase in BP might be related to the continuous increase in body weight or incomplete RDN or reinnervation of the kidneys. Of note, compared with the control group, kidney NEPI concentrations were reduced significantly and there were no signs of reinnervation in the histopathological analyses. The pigs treated herein did not receive antihypertensive medication. Therefore, it remains to be proven if renal renin AV-Δ is influenced by concomitant antihypertensive medication. As the blood sampling from the renal artery and vein before and after the HC required anesthesia, the HC was only done at baseline and termination. Thus, the observed changes in renal renin AV-Δ might also be attributed, in part, to weight gain, metabolic changes, different baseline renin values, and ageing in the treated animals independently of RDN. Furthermore, we cannot exclude that altered renal autoregulation has affected renal renin AV-Δ in these very unhealthy animals. However, creatinine, a marker for renal function, remained unchanged during the study.

## Conclusion

RDN, using a multielectrode RF catheter, reduced renal NEPI tissue level and weight-adjusted BP. Following RDN, HC-induced renal renin AV-Δ was attenuated. Importantly, a higher renal renin AV-Δ at baseline was associated with a more pronounced BP reduction at follow-up. Renal renin AV-Δ at baseline and during an HC should be investigated in human hypertension to assess the approach’s suitability for patient selection and procedural guidance.

## Disclosures

LBM, CAP, JMF, and JAJ are employees of Abbott, Inc. MB has received honoraria for lectures and scientific advice from Abbott, Astra-Zeneca, Boehringer-Ingelheim, Medtronic, Servier, and Vifor. FM is supported by Deutsche Gesellschaft für Kardiologie (DGK), FM and MB are supported by Deutsche Forschungsgemeinschaft (SFB TRR219). SE and FM have received scientific support and speaker honoraria from Medtronic and ReCor Medical. The remaining authors have nothing to disclose.

## Electronic supplementary material

Below is the link to the electronic supplementary material.

Supplementary file1 (DOCX 17 kb)
