# Supplementary material for: A drug-induced hypotensive challenge to verify catheter-based radiofrequency renal denervation in an obese hypertensive swine model
Source: Clin Res Cardiol. 2020 Nov 2;111(6):595–603. doi: 10.1007/s00392-020-01764-0 (PMC9151536; doi:10.1007/s00392-020-01764-0)
Supplement: Supplementary file 1 — Supplementary file1 (DOCX 17 kb) [file 392_2020_1764_MOESM1_ESM.docx]

**Supplemental material**

**Supplemental table S1. Renin concentration in the renal arteries and veins**

| **Animal** | **Before RDN** | | | | **After RDN** | | | |
| --- | --- | --- | --- | --- | --- | --- | --- | --- |
|  | **Pre-HC** | | **Post-HC** | | **Pre-HC** | | **Post-HC** | |
|  | **Arterial**  **(pg/mL)** | **Venous**  **(pg/mL)** | **Arterial**  **(pg/mL)** | **Venous**  **(pg/mL)** | **Arterial**  **(pg/mL)** | **Venous**  **(pg/mL)** | **Arterial**  **(pg/mL)** | **Venous**  **(pg/mL)** |
| 01 | 1.511 | 0.692 | 1.172 | 0.801 | 0.886 |  |  |  |
| 02 | 1.221 | 1.305 | 1.197 | 1.612 | 0.936 | 0.936 | 0.986 | 1.036 |
| 03 | 1.780 | 0.804 |  | 0.750 | 0.936 | 0.936 | 0.936 | 1.036 |
| 04 | 1.480 | 1.418 | 0.783 | 1.407 | 0.736 | 0.886 | 0.886 | 0.936 |
| 05 | 1.240 | 0.835 | 1.386 | 1.454 | 0.936 | 0.936 | 1.036 | 0.986 |
| 06 | 1.644 | 0.736 | 0.685 | 0.890 | 1.036 |  |  |  |
| 07 | 1.707 | 0.636 | 0.772 | 0.870 | 0.836 | 0.986 | 0.886 | 1.036 |
| 08 | 0.828 | 1.196 | 0.952 | 0.850 |  |  |  |  |
| 09 | 0.748 | 0.987 | 0.799 | 0.906 |  |  |  |  |
| 10 | 1.187 | 1.314 | 0.824 | 1.358 | 1.036 | 0.936 | 1.136 | 0936 |
| 11 | 1.240 | 1.276 | 1.048 | 1.340 | 0.936 | 1.136 | 1.136 | 0.936 |
| 12 | 1.024 | 0.988 | 1.191 | 1.194 |  |  |  |  |

**Supplemental table S2: Distribution of renal nerves stratified according to distance from the lumen in relative proximal, middle, and distal location and distance from nerve to the lumen of the renal artery (five renal arteries)**

|  | **Overall** | **Distance from lumen to nerve** | | | | **p-value^a^** |
| --- | --- | --- | --- | --- | --- | --- |
|  |  | **≤2 mm** | **2-4 mm** | **4-6 mm** | **>6 mm** |  |
| Proximal segment, n (%) | 201(100) | 36 (18) | 42 (21) | 30 (15) | 93 (46) | <0.0001 |
| Middle segment, n (%) | 126 (100) | 79 (63) | 23 (18) | 14 (11) | 10 (8) |  |
| Distal segment, n (%) | 385 (100) | 256 (66) | 75 (19) | 20 (5) | 34 (9) |  |
| Post-bifurcation, n (%) | 551 (100) | 401 (73) | 83 (15) | 42 (8) | 25 (5) |  |
| **Nerves ≥100 µm in diameter** | | | | | | |
| Proximal segment, n (%) | 38 (100) | 2 (5) | 5 (13) | 5 (13) | 26 (68) | <0.0001 |
| Middle segment, n (%) | 26 (100) | 14 (54) | 7 (27) | 3 (12) | 2 (8) |  |
| Distal segment, n (%) | 104 (100) | 60 (58) | 28 (27) | 7 (7) | 9 (9) |  |
| Post-bifurcation, n (%) | 130 (100) | 100 (77) | 18 (14) | 9 (7) | 3 (2) |  |
| **Nerves ≥200 µm in diameter** | | | | | | |
| Proximal segment, n (%) | 9 (100) | 0 (0) | 1 (11) | 0 (0) | 8 (80) | <0.0001 |
| Middle segment, n (%) | 11 (100) | 5 (45) | 5 (45) | 1 (9) | 0 (0) |  |
| Distal segment, n (%) | 28 (100) | 18 (64) | 7 (25) | 2 (7) | 1 (4) |  |
| Post-bifurcation, n (%) | 51 (100) | 41 (80) | 6 (12) | 3 (6) | 1 (2) |  |

Values are numbers (%). ^a^p-value calculated using the Kruskal-Wallis test with the distance as the grouping factor.

**Supplemental table S3: Minimal diameter of nerves stratified according to distance from the nerve to the lumen of the renal artery (five renal arteries)**

|  | **Overall** | **Distance from lumen to nerve** | | | | **p-value^a^** |
| --- | --- | --- | --- | --- | --- | --- |
|  |  | **≤2 mm** | **2-4 mm** | **4-6 mm** | **>6 mm** |  |
| **Distance from ostium** | | | | | | |
| Proximal segment, µm | 64.0±57.8 | 39.4±35.5 | 54.4±43.8 | 59.1±49.6 | 79.3±68.1 | 0.002 |
| Middle segment, µm | 70.9±78.1 | 65.3±78.9 | 92.5±89.3 | 72.4±67.5 | 64.1±55.5 | 0.527 |
| Distal segment, µm | 74.4±70.7 | 68.1±69.8 | 94.9±78.6 | 86.3±55.9 | 69.6±58.9 | 0.028 |
| Post-bifurcation, µm | 77.7±79.9 | 80.3±83.6 | 75.3±76.1 | 74.7±64.0 | 49.2±45.8 | 0.289 |
| **Circumferential distribution** | | | | | | |
| Anterior quadrant, µm | 80.4±83.7 | 79.4±86.9 | 95.3±91.0 | 64.4±73.0 | 64.4±73.0 | 0.422 |
| Posterior quadrant, µm | 58.0±58.2 | 55.6±58.0 | 54.3±48.6 | 84.3±67.9 | 84.3±67.9 | 0.192 |
| Superior quadrant, µm | 79.4±74.8 | 79.3±81.5 | 83.1±72.1 | 73.3±64.6 | 73.3±64.6 | 0.933 |
| Inferior quadrant, µm | 72.1±72.6 | 76.3±79.9 | 70.0±67.1 | 49.4±48.6 | 62.7±48.6 | 0.399 |

Values are mean ± standard deviation (SD). ^a^p-value calculated using ANOVA.
